# Influence of anaemia in severely injured patients on mortality, transfusion and length of stay: an analysis of the TraumaRegister DGU^®^

**DOI:** 10.1007/s00068-021-01869-9

**Published:** 2022-01-20

**Authors:** Linda Tanner, Vanessa Neef, Florian J. Raimann, Philipp Störmann, Ingo Marzi, Rolf Lefering, Kai Zacharowski, Florian Piekarski, Committee on Emergency Medicine, Intensive Care and Trauma Management (Sektion NIS) of the German Trauma Society (DGU)

**Affiliations:** 1 Department of Anaesthesiology, Intensive Care Medicine and Pain Therapy, University Hospital Frankfurt, Goethe University, Frankfurt, Theodor-Stern-Kai 7, 60590 Frankfurt am Main, Germany; 2 Department of Trauma, Hand and Reconstructive Surgery, University Hospital Frankfurt, Goethe-University, Frankfurt, Germany; 3 Faculty of Health, University of Witten/Herdecke, Witten, Germany; 4 IFOM, Institute for Research in Operative Medicine, Faculty of Health, University Witten/Herdecke, Cologne, Germany; 5 Committee on Emergency Medicine, Intensive Care and Trauma Management (Sektion NIS) of the German Trauma Society (DGU), Berlin, Germany

**Keywords:** Anaemia, Polytrauma, Outcome, Mortality, Trauma centre, Transfusion, TraumaRegister DGU^®^

## Abstract

**Purpose:**

Anaemia is one of the leading causes of death among severely injured patients. It is also known to increase the risk of death and prolong the length of hospital stay in various surgical groups. The main objective of this study is to analyse the anaemia rate on admission to the emergency department and the impact of anaemia on in-hospital mortality.

**Methods:**

Data from the TraumaRegister DGU^®^ (TR-DGU) between 2015 and 2019 were analysed. Inclusion criteria were age ≥ 16 years and most severe Abbreviated Injury Scale (AIS) score ≥ 3. Patients were divided into three anaemia subgroups: no or mild anaemia (NA), moderate anaemia (MA) and severe anaemia (SA). Pre-hospital data, patient characteristics, treatment in the emergency room (ER), outcomes, and differences between trauma centres were analysed.

**Results:**

Of 67,595 patients analysed, 94.9% (*n* = 64,153) exhibited no or mild anaemia (Hb ≥ 9 g/dl), 3.7% (*n* = 2478) displayed moderate anaemia (Hb 7–8 g/dl) and 1.4% (n = 964) presented with severe anaemia (Hb < 7 g/dl). Haemoglobin (Hb) values ranged from 3 to 18 g/dl with a mean Hb value of 12.7 g/dl. In surviving patients, anaemia was associated with prolonged length of stay (LOS). Multivariate logistic regression analyses revealed moderate (*p* < 0.001 OR 1.88 (1.66–2.13)) and severe anaemia (*p* < 0.001 OR 4.21 (3.46–5.12)) to be an independent predictor for mortality. Further significant predictors are ISS score per point (OR 1.0), age 70–79 (OR 4.8), age > 80 (OR 12.0), severe pre-existing conditions (ASA 3/4) (OR 2.26), severe head injury (AIS 5/6) (OR 4.8), penetrating trauma (OR 1.8), unconsciousness (OR 4.8), shock (OR 2.2) and pre-hospital intubation (OR 1.6).

**Conclusion:**

The majority of severely injured patients are admitted without anaemia to the ER. Injury-associated moderate and severe anaemia is an independent predictor of mortality in severely injured patients.

## Introduction

Anaemia remains one of the leading causes of death in severely injured patients [1]. It can be both acute, caused by massive haemorrhage, and/or chronic at the time of admission [2]. Besides trauma-related coagulopathy, hypothermia, haemodilution and shock, anaemia therapy represents a key aspect in the treatment of severely injured patients. Furthermore, anaemia impacts patients’ clinical outcomes and contributes to the costs associated with trauma [3]. Acute anaemia often requires the transfusion of red blood cells (RBCs) [4]. However, blood transfusions have shown to be an independent predictor for mortality, length of stay (LOS) in the intensive care unit (ICU) and systemic inflammatory response syndrome (SIRS) [5, 6].

The spectrum of patients, the outcome in trauma patients and the mechanism of trauma are diverse, ranging from common head and thoracic injuries to abdominal and pelvic injuries [7]. Patients with combined injuries or without head involvement tend to be younger than patients with isolated head injuries [7]. Elderly trauma patients more often present with lower haemoglobin (Hb) levels in the emergency room (ER) and at discharge and more frequently receive transfusions of RBCs than younger patients [8]. Furthermore, the severity of anaemia at admission predicted 6-month mortality in geriatric patients [9]. Initial and lowest Hb after admission additionally predicted outcomes in patients with traumatic brain injuries [10]. Admission procedures take into account the particular importance of anaemia in severely injured patients via several prognostic scores, e.g. the 'Revised Injury Severity Classification' (RISC) II score [11].

This TraumaRegister DGU^®^ (TR-DGU) study analyses the incidence of anaemia and effects of anaemia on in-hospital mortality as well as on red blood cell transfusion and length of hospital stay. Further possible factors influencing anaemia will be analysed.

The main objective of this study is to analyse the anaemia rate on admission to the emergency department and the impact of anaemia on in-hospital mortality. In addition, the study also investigates the transfusion rate and in-hospital outcomes of critically injured patients.

## Materials and methods

The TraumaRegister DGU^®^ of the German Trauma Society (Deutsche Gesellschaft für Unfallchirurgie, DGU) was founded in 1993. The aim of this multi-centre database is the pseudonymised and standardised documentation of severely injured patients [12]. Participating hospitals are located in Germany (90%), Belgium, Finland, Luxembourg, the Netherlands, Austria, Switzerland, Slovenia and the United Arab Emirates.

The register documents data from (1) the pre-hospital phase, (2) the ER, (3) ICU stays and (4) hospital discharge, including detailed information on patient demographics, the mechanism of trauma, comorbidities, pre- and in-hospital management, the course of treatment in the ICU, relevant laboratory findings, such as transfusion data, and final patient outcomes. Every patient whose ER admission results in death or a stay at the ICU of a participating trauma centre is included in the database.

The infrastructure for documentation, data management and data analysis are provided by the ‘AUC—Academy for Trauma Surgery’, a company affiliated with the DGU. Scientific leadership is provided by the DGU’s ‘Committee on Emergency Medicine, Intensive Care and Trauma Management (Sektion NIS)’. Scientific data analysis is approved according to a peer review procedure outlined in the publication’s guidelines for the TR-DGU. Approximately, 30,000 cases from more than 650 hospitals are currently registered into the database annually.

Participation in the TR-DGU is voluntary. However, for hospitals associated with TraumaNetzwerk DGU^®^, the entry of at least a basic data set is mandatory as part of the quality management programme.

The present study is in line with the publication guidelines of the TraumaRegister DGU^®^ and registered under the TR-DGU project ID: 2020–054. The study was performed in accordance with the Declaration of Helsinki.

### Inclusion criteria

Patients admitted to a German ER from 2015 to 2019 were analysed for this study. Inclusion criteria were age ≥ 16 years and Abbreviated Injury Scale (AIS) score ≥ 3. The Abbreviated Injury Scale (AIS) is an anatomically based injury severity rating system that classifies each injury by body region on a six-point scale (AIS 1—minor up to AIS 6—maximum). Patients were classified into three categories of anaemia: (1) no or mild anaemia (NA; Hb ≥ 9 g/dl), (2) moderate anaemia (MA; Hb 7–8 g/dl) and (3) severe anaemia (SA; Hb < 7 g/dl). The registry does not collect any chronic anaemia parameters; therefore, patients with low Hb values (Hb < 9 g/dl) but normal blood pressure (> 110 mmHg) and only minor pre-hospital volume therapy (< 1000 mL) were defined as chronically anaemic. Transfusion rate was defined as a minimum of 1 transfusion of an RBC unit per patient in relation to the total number of patients in the ER.

**Fig. 1:**
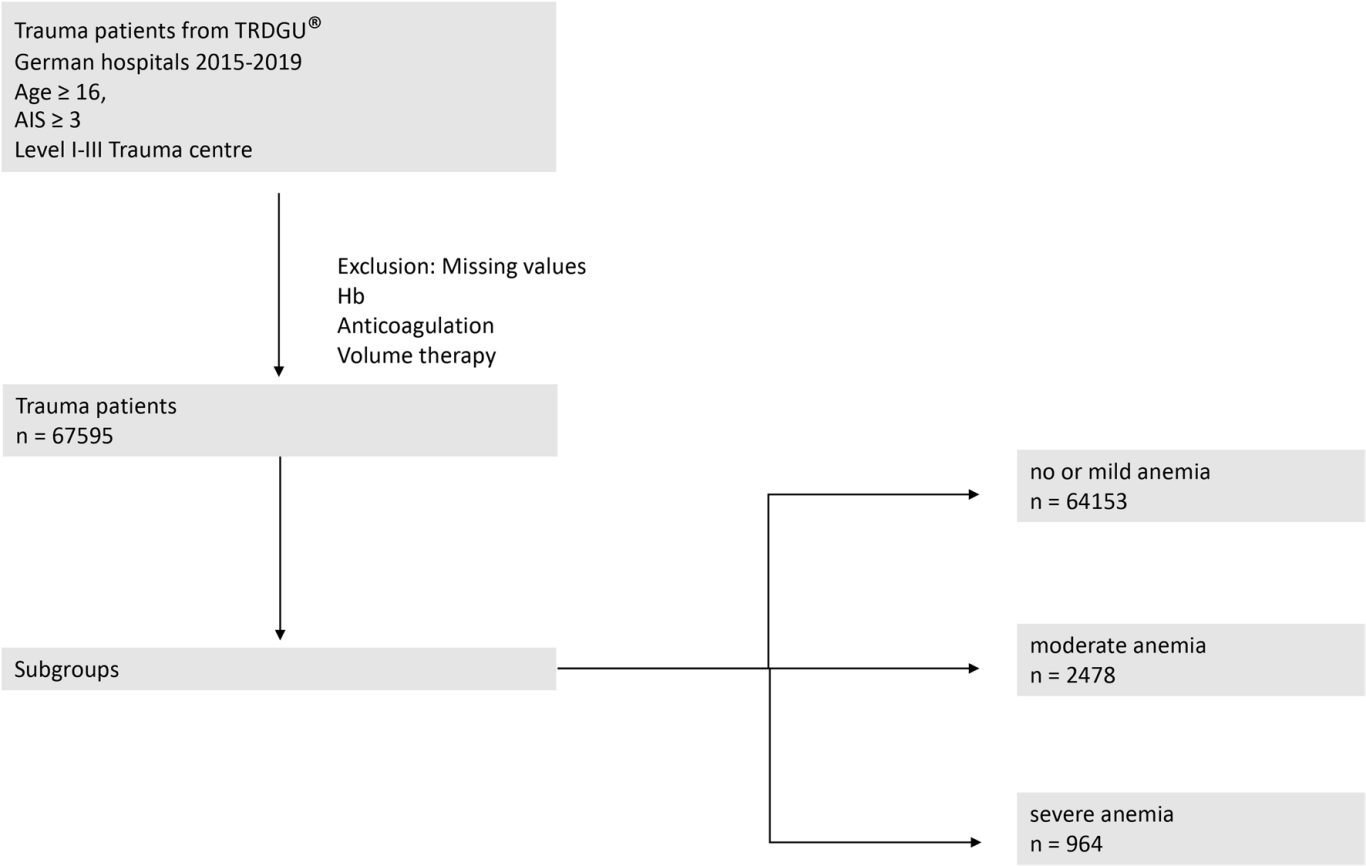
Flowchart for patient inclusion. Patients (aged ≥ 16 years) for primary analysis were included with an Abbreviated Injury Scale (AIS) of ≥ 3 who were directly admitted to a German trauma centre (TraumaZentrum DGU^®^) from 2015 to 2019. Patients were divided into anaemia subgroups (no or mild anaemia Hb ≥ 9 g/dl, moderate anaemia Hb 7–8 g/dl, severe anaemia Hb < 7 g/dl). *AIS* Abbreviated Injury Scale, *Hb* haemoglobin

Patients who were transferred during treatment were excluded because of missing data from the pre-hospital phase (transfer in cases) or missing hospital outcome (transfer out cases), respectively. To facilitate comparisons, patients with missing values for Hb (2.1%) or pre-hospital volume therapy (9.2%) were also excluded (Fig. 1).

### Statistical analysis

Anaemia at admission to the ER, transfusion of RBCs, volume therapy, anticoagulation, mechanism of trauma, ICU stay, cause of death, pre-hospital time of rescue, and general fatality were analysed. Possible factors influencing anaemia are evaluated descriptively. These include pre-hospital volume therapy, haemodynamic situation, intake of anticoagulation, injury severity, and injury pattern. In the register, the following drugs are recorded as anticoagulants: acetylsalicylic acid (ASS), vitamin K antagonists, new oral anticoagulants (NOACs) and heparin.

Multivariable logistic regression analysis was performed with hospital mortality as dependent variable. The variables were adapted from the RISC-2 score. Besides anaemia, the following variables were included as potential predictors (independent variables): age, sex, pre-injury disease, Injury Severity Score, head injury, penetrating trauma, unconsciousness, shock, pre-hospital intubation and volume therapy, and hospital level of care.

The predictability of mortality on the basis of the RISC-2 score, a 13-factor prognostic score for mortality, as a function of the severity of anaemia will be assessed in a multivariate model.

Statistical analysis was performed using SPSS Statistics (Version 24, IBM, Armonk, New York). Descriptive results are presented as counts and percentages for categorical data, and as mean with standard deviation (SD) or as median with inter-quartile range (IQR) for metric data, respectively. Resulting odds ratios are presented with 95% confidence intervals (CI). A *p* value below 0.05 was considered significant. Illustrations were created with Prism (Version 9.1.0 for macOS, GraphPad Software, San Diego).

## Results

A total of 67,595 severely injured patients (AIS ≥ 3) were analysed between 2015 and 2019. The demographic data and mechanism of trauma are shown in Table 1.

**Table 1:** Demographics, anaemia, transfusion, outcomes and mechanism of trauma

	No or mild anaemia (NA) 64,153 [*n* (%)]	Moderate anaemia (MA) 2478 [*n* (%)]	Severe anaemia (SA) 964 [*n* (%)]
Demographics			
Female	18,631 (92.4%)	1103 (5,4%)	424 (2.1%)
Male	45,522 (96.0%)	1375 (2.9%)	540 (1.1%)
< 60 years	36,168 (95.8%)	1100 (2.9%)	493 (1.3%)
≥ 60 years	27,985 (93.8%)	1378 (4.6%)	471 (1.6%)
ASA > 3 12,641	11,580 (91.6%)	785 (6.2%)	276 (2.2%)
ISS	19.9 ± 10.3	28.9 ± 16.0	33.6 ± 17.9
Anaemia and transfusion			
Acute anaemia	Not applicable	1742 (69.1%)	780 (30.9%)
Chronic anaemia	Not applicable	666 (80.9%)	157 (19.1%)
Hb g/dl mean	13.4 ± 1.8	8.1 ± 0.6	5.8 ± 1.0
RBC transfusion in ER	3710 (70.2%)	971 (18.4%)	604 (11.4%)
> 10 RBC Units in ER	377 (55.0%)	178 (26.0%)	130 (19.0%)
RBC Units in ER	0.27 ± 1.7	2.5 ± 5.5	4.8 ± 8.2
Trauma-associated severe haemorrhage score	3.9 ± 3.4	12.8 ± 4.4	16.6 ± 4.7
Fresh frozen plasma in ER	2180 (72.4%)	513 (17.0%)	320 (10.6%)
Thrombocytes in ER	527 (63.6%)	182 (22.0%)	119 (14.4%)
PCC in ER	1295 (73.5%)	297 (16.9%)	169 (9.6%)
Fibrinogen in ER	5105 (84.0%)	651 (10.7%)	324 (5.3%)
Tranexamic acid PH	4617 (87.1%)	446 (8.4%)	238 (4.5%)
Volume therapy PH (ml)	695 ± 526	1098 ± 854	1386 ± 1107
Volume therapy ER (ml)	1113 ± 1435	1975 ± 2288	2528 ± 2660
Anticoagulation			
ASS	6100 (94.2%)	288 (4.4%)	86 (1.3%)
Direct oral anticoagulants	2224 (92.0%)	147 (6.1%)	47 (1.9%)
Vitamin K antagonists	2365 (92.8%)	131 (5.1%)	52 (2.0%)
Haemodynamics			
Shock (syst. BP ≤ 90 mmHg) in ER	4330 (80.8%)	675 (12.6%)	354 (6.6%)
RR Syst (mmHg) PH	136.5 ± 32.1	113.3b ± 42.2	98.2 ± 47.0
RR Syst (mmHg) in ER	136.1 ± 29.5	110.6 ± 39.1	94.1 ± 44.3
Vasopressors in ER	4750 (83.0%)	632 (11.0)	344 (6.0%)
CPR in ER	459 (62.4%)	135 (18.4%)	141 (19.2%)
Outcome			
Mortality	7181 (83.3%)	910 (10.6%)	533 (6.2%)
RISC II score	10.2%	34.0%	55.3%
Dead in ER	471 (58.0%)	178 (21.9%)	163 (20.1%)
Dead in 24 h	3191 (76.9%)	570 (13.7%)	393 (9.5%)
Stay in ICU (days)	6.2 ± 9.9	10.6 ± 16.3	9.0 ± 24.6
Length of in-hospital stay	16.0 ± 16.2	19.8 ± 24.4	16.6 ± 24.6
Multiorgan dysfunction	5429 (87.8%)	517 (8.4%)	238 (3.8%)
Sepsis	1721 (89.2%)	140 (7.3%)	69 (3.6%)
Mechanism and trauma			
Head injury	9706 (96.3%)	297 (2.9%)	80 (0.8%)
Combined head injury^1^	22,518 (93.5%)	1117 (4.6%)	460 (1.9%)
GCS < 8	9943 (87.8%)	904 (8.0%)	480 (4.2%)
Thorax (AIS ≥ 3)	31,484 (94.3%)	1344 (4.0%)	558 (1.7%)
Abdomen (AIS ≥ 3)	6905 (89.3%)	523 (6.8%)	306 (4.0%)
Extremities (AIS > = 3)	18,606 (91.9%)	1143 (5.6%)	493 (2.4%)
Traffic accident by car	12,945 (95.0%)	476 (3.5%)	201 (1.5%)
Traffic accident by motorcycle	8919 (96.0%)	257 (2.8%)	118 (1.3%)
Traffic accident by bicycle	6237 (97.1%)	132 (2.1%)	53 (0.8%)
Traffic accident by pedestrian	3488 (92.0%)	213 (5.6%)	90 (2.4%)
Fall > 3 m	9631 (94.7%)	386 (3.8%)	148 (1.5%)
Fall < 3 m	16,589 (95.0%)	681 (3.9%)	200 (1.1%)
Traffic accident other	1008 (90.7%)	77 (6.9%)	26 (2.3%)
Explosion	1830 (96.8%)	43 (2.3%)	18 (1.0%)
Shot	301 (87.2%)	30 (8.7%)	14 (4.1%)
Stab	1190 (88.5%)	103 (7.7%)	51 (3.8%)
Other mechanism	1502 (94.3%)	57 (3.6%)	34 (2.1%)
Rescue time			
< 40 min	9528 (96.1%)	291 (2.9%)	100 (1%)
40–90 min	38,204 (95.2%)	1371 (3.4%)	542 (1.4%)
> 91 min	8513 (92.7%)	472 (5.1%)	195 (2.1%)

The percentage in each group (SA, MA, NA) is given in brackets (%). The total *n* per item may differ because individual items are documented with different frequency

^1^Combined head injury = head injury and ≥ 1 other injury pattern (e.g. abdominal or thoracic trauma)

The mean ISS was 19.9 ± 10.3 in the non-anaemic, 28.9 ± 16.0 in the moderately anaemic and 33.6 ± 17.9 in the severely anaemic group. Trauma-related major bleeding score was higher with increasing anaemia level (NA 3.9 ± 3.4, MA 12.8 ± 4.4, SA 16.6 ± 4.7).

### Anaemia and transfusion

**Fig. 2:**
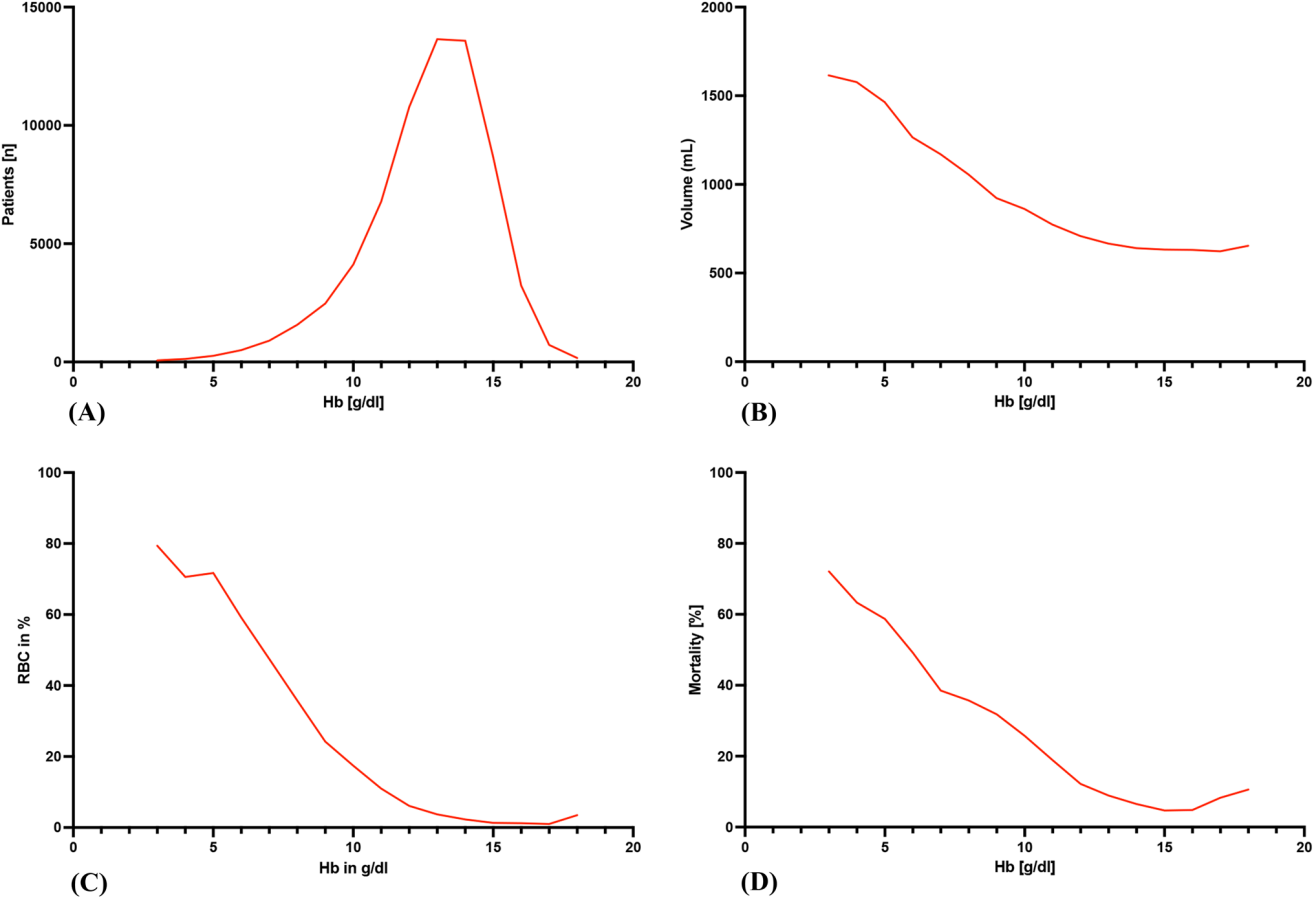
Correlation of haemoglobin concentration and mean pre-hospital volume, blood transfusion rate and mortality. **A** Distribution of Hb values at admission are illustrated. Mean Hb value at admission was 12.66 g/dl. Hb values ≤ 3 g/dl and ≥ 18 g/dl were pooled for means of comparison. **B** Distribution of pre-hospital volume therapy is illustrated. Pre-hospital volume therapy ranges from 500 to 1500 mL. **C** Distribution of RBC transfusion in ER rate related to Hb values is illustrated. The rate of RBC transfusion increases exponentially with decreasing Hb values. **D** Mortality rate associated with Hb value is illustrated. The mortality rate rises exponentially with decreasing Hb values. *Hb* haemoglobin, *RBC* red blood cell

Among the severely injured patients in the study, 64,153 (94.9%) had no or mild anaemia (Hb ≥ 9 g/dl), 2,478 (3.7%) presented with moderate anaemia (Hb 7–8 g/dl) and 964 (1.4%) patients exhibited severe anaemia (Hb < 7 g/dl). Haemoglobin values ranged from 3 to 18 g/dl with a mean Hb value of 13.1 ± 2.2 g/dl (Fig. 2a). Among all patients, 823 (1.2%) had chronic anaemia, and 2522 had acute anaemia (3.7%).

In total, 7.8% (5285) of patients were transfused. Severe anemia is associated with a higher transfusion rate (65.4%) than MA (40.0%) and NA (5.8%). Among female patients, the proportion of MA was higher (5.5%) than among male patients (2.9%), likewise the proportion of SA was higher (2.1%) than among male patients (1.1%). The highest rates of MA and SA are found in shooting injuries (MA 8.7%, SA 4.1%), stabbing injuries (MA 7.7%, SA 3.8%), and traffic accidents by pedestrian (MA 5.6%, SA 2.4%) (Table 1).

Massive transfusions of more than ten red cell units per patient were recorded in the context of severe anaemia at 14.1%, compared to 0.6% for NA and 7.3% for MA. RBC transfusion rate in ER increases with lower Hb values reaching a maximum in patients with Hb values of 4 g/dl and a minimum in patients with Hb values of 15–17 g/dl (Fig. 2C). Analysis showed that patients with severe anaemia (42.3%) were more likely to suffer from haemodynamic instability, defined by a systolic blood pressure below 90 mmHg, than patients with no (7.1%) or moderate anaemia 30.0%). Patients with severe anaemia received on a mean basis more fluid therapy in the pre-hospital and shock room phase compared to the other groups (Table 1). Pre-hospital values for volume therapy ranged from 500 mL for patients with high Hb values to a maximum of 1500 mL for patients with Hb values < 5 g/dl (Fig. 2B).

### Outcome

**Fig. 3:**
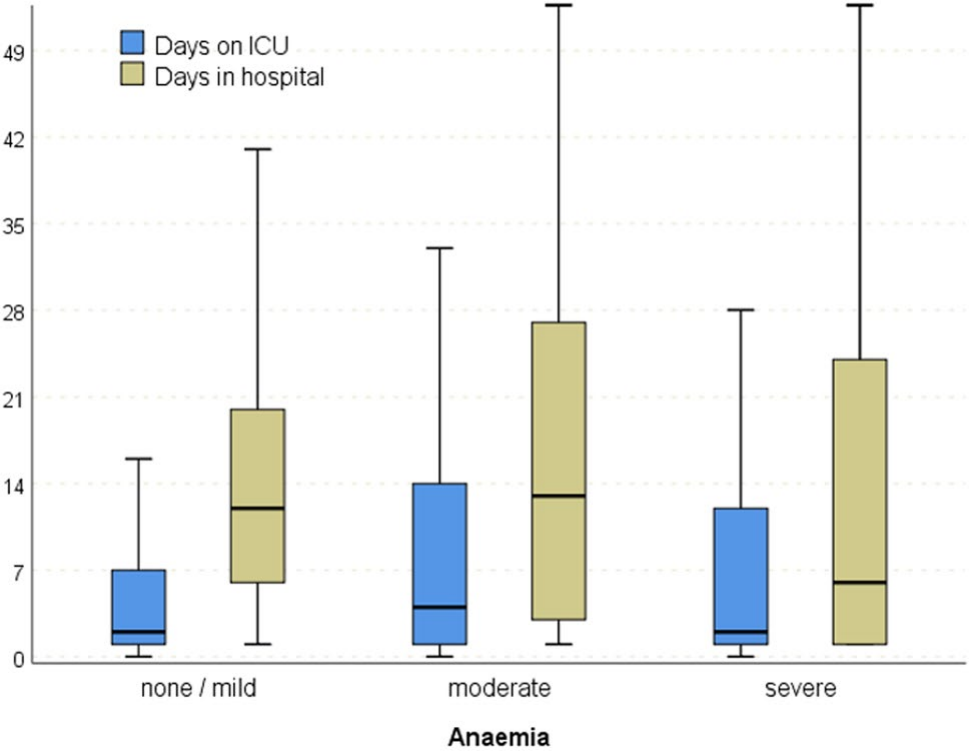
Length of hospital and ICU stay dependence of anaemia severity. Length of hospital stay for surviving patients. Length of hospital stay was associated with severity of anaemia

Multivariate logistic regression analyses revealed moderate (*p* < 0.01 OR 1.88 (1.66–2.13) and severe anaemia (*p* < 0.01 OR 4.21 (3.46–5.12) to be an independent risk factor for higher mortality (Table 2). Thus, mortality rate increases with severity of anaemia, ranging from 4.7% in NA patients to 72.1% in SA patients (Fig. 2D). Patients with SA died most often in the ER (17.0%) and within 24 h after admission to ER (23.0%), while patients with MA most often died within 24 h after admission to ER (15.6%). Multivariable logistic regression analyses revealed that age, pre-existing diseases, ISS score per point, severity of head injury (AIS > 4), shock, unconsciousness, and anaemia are predictors for higher mortality (Table 2). The group with moderate anaemia had the longest mean length of stay in hospital (19.8 ± 24.4 days) and the longest length of stay in intensive care (10.6 ± 16.4 days). Shorter lengths of stay are seen in severe anaemia (ICU 9.0 ± 15.1 and LOS 17.6 ± 24.6 and in those without anaemia (ICU 16.0 ± 16.2 and LOS 6.2 ± 9.9 days) (Fig. 3).

**Table 2:** Multivariate analysis of independent predictors for mortality

Predictor	Value	*p* value	Odds ratio	95% CI for OR
Age (years) (reference age: 16–59 years)	60–69	< 0.001	2.33	2.09–2.60
	70–79	< 0.001	4.84	4.38–5.34
	80+	< 0.001	12.04	10.89–13.30
Sex	Males	0.007	1.10	1.03–1.18
Pre-existing diseases	ASA 3/4	< 0.001	2.26	2.09–2.43
Injury Severity Score	per point	< 0.001	1.04	1.04–1.04
Head injury severity (reference: AIS 0–2)	AIS 3	0.632	0.98	0.88–1.08
	AIS 4	< 0.001	1.29	1.17–1.42
	AIS 5/6	< 0.001	4.76	4.32–5.23
Penetrating trauma	Yes	< 0.001	1.80	1.52–2.15
Unconscious (GCS 3–8)	Yes	< 0.001	4.83	4.40–5.31
Shock (syst. BP ≤ 90 mmHg)	Yes	< 0.001	2.19	2.01–2.38
Pre-hospital intubation	Yes	< 0.001	1.63	1.48–1.79
Pre-hospital volume therapy	≥ 1000 ml	0.149	0.93	0.84–1.03
Level of care (reference: Level 1)	Level 2	0.033	1.09	1.01–1.17
	Level 3	0.253	0.92	0.80–1.06
Anaemia (reference: no or mild anaemia)	Moderate anaemia	< 0.001	1.88	1.66–2.13
	Severe anaemia	< 0.001	4.21	3.46–5.12

RISC II scores offer an adequate prognosis of death in patients with NA and SA, but fail to predict death in patients with MA. A multivariable analysis reveals that the same results can be applied to the three levels of trauma centres (Level I–III centres). RISC II scores modelled mortality equally well for each trauma centre. Across levels, the outcomes for NA and SA patients were well estimated by RISC II scores.

## Discussion

In this study, we revealed that in-hospital mortality and transfusion rates increased with the severity of anaemia and that anaemia was associated with prolonged Hospital-LOS [13]. Multivariable analyses revealed that severe and moderate anaemia are a risk factor for higher mortality.

Among trauma patients, anaemia remains one of the most common causes of death upon admission to the ER [1]. Massive haemorrhage due to severe trauma can cause acute anaemia. Severe anaemia in trauma patients is a consequence of a sustained severe injury, yet there was a higher OR for severe anaemia (OR 4.21) compared to other predictors such as shock, defined as systolic BP ≤ 90 mmHg, (OR 2.19), or penetrating trauma (OR 1.80). SA had a similar OR to severe head injury (AIS 5/6: OR 4.76), age over 70 years (OR 4.84–12.04) or unconsciousness at the scene (OR 4.83). These results are in line with the findings of Lefering et al., who also showed a significant influence (*p* < 0.001 OR 1.45) of traumatic anaemia on mortality with a haemoglobin limit value of less than 12 g/dl [11]. Froessler et al. revealed that anaemia increases mortality, LOS and costs associated with trauma management [3].

In the emergency setting, treatment usually involves RBC transfusions. RBC transfusions, in turn, are associated with greater mortality, an increased risk for SIRS and prolonged ICU and hospital stays [5, 6].

In summary, 67,595 injured patients were analysed. Both the mean Hb values and the distribution of Hb values from trauma patients are comparable to the German population in general [14]. Although this study included only severely injured patients with AIS scores ≥ 3, the majority of patients were admitted without anaemia to the ER. Pre-clinically applied volume resuscitation has an influence on patients’ Hb concentrations at the time of ER admission [15]. Pre-hospital mean volume resuscitation ranged from 500 mL for patients with high Hb values to a maximum of 1.5 L for patients with Hb values < 5 g/dl. These findings align with the current guidelines for pre-hospital trauma management [16]. According to data from the TraumaRegister DGU^®^, volume resuscitation has become more restrictive during the last decade [15]. Our analysis demonstrated that patients with SA received a maximum of 1500 mL crystalloid volume, minimising the risk for haemodilution and coagulopathy. Thus, it can be assumed that the determined Hb values used for this analysis are both realistic and caused by haemorrhage and not by haemodilution.

The RBC transfusion rate in anaemic patients increased exponentially with decreasing Hb values. Patients with an Hb value of 8 g/dl showed signs of chronic anaemia in 40% of all registered cases, a finding that reflects the current prevalence of chronic anaemia in adults. Chronic anaemia is present in 17% of adults over 60 years and in 40% of elderly patients [17]. Because anaemia is associated with functional restrictions, limited mobility and decreased stability and balance, moreover, it also increases the risk for falls [18]. Laboratory values that are indicative of chronic anaemia, such as transferrin saturation, are not collected in the registry. Therefore, prospective studies are needed to further investigate the influence of chronic anaemia in trauma patients.

We found that anticoagulation in severely injured patients seemed not to be associated with anaemia. Compared to anaemic trauma patients, all trauma patients in our analysis had a similar distribution of anticoagulant medication. Neither of the different anticoagulation groups was associated with a greater risk for anaemia at admission. This result is surprising since the use of antiplatelet medications or anticoagulation is associated with an increased bleeding tendency and coagulopathy. It also increases the importance of point-of-care coagulation testing (POCT) in the ER. Based on the available data in this analysis, it shows that in severely injured patients with severe anaemia, POCT diagnostics were performed in 12.4% of cases. ASS impacts thrombocyte function, which can be rapidly diagnosed with viscoelastic measurements in the ER or during surgery [19]. Point-of-care analyses, such as viscoelastic measurements, platelet function tests or blood gas analysis, can enable the rapid detection of massive bleeding and, due to their quicker results and the smaller sample sizes required, should be favoured to laboratory testing. With a rapid turnaround time and minimal sample size, blood gas analysis has proven to be a valid measurement method for Hb values in the ER [20]. POCT must be part of patients’ blood management, ultimately improving the patient outcomes [21]. Due to the lack of haemodilution, results from this testing can be safely used for clinical decision-making.

Several studies have already demonstrated the increased mortality of anaemic patients [1]. Our study has reproduced these results for a cohort of severely injured patients. In our study, however, patients with SA tended to die rather quickly—in the ER or during the first 24 h after injury, while patients with MA more often survived the acute phase in the ER but died during emergency surgery or within 24 h of admission to the ICU. This raises the question of why patients with severe anaemia do not survive the ER despite the possibility of immediate massive transfusion. Thus, mass transfusion protocols have been established in trauma centres to ensure rapid and correct decisions in bleeding situations. However, it is known that massive haemorrhage can be treated specifically but can lead to several complications such as organ dysfunction or coagulopathy [22]. Considering the presumed causes of death of patients with severe anaemia, 40.6% were traumatic brain injury, 31.2% haemorrhage, 20.5% organ failure, and 7.7% other causes. This also shows in our analysis that severe haemorrhage is the presumed cause of death with almost one-third of the patients. As a recently published review points out, several factors need to be considered in the treatment of traumatic haemorrhage: early haemorrhage control, resuscitation efforts, and a more complete understanding of the pathophysiology of coagulopathy in trauma, sepsis, and MOF may lead to further reduction of mortality in trauma-induced haemorrhage [23].

### Limitations

This is a retrospective analysis of the TraumaRegister DGU^®^. Because registered data are less valid than data taken from a prospective randomised study, the results we observed should be seen only as associations and not as causations. Finally, because the database we employed is generated by medical personnel, it is vulnerable to human bias. The definition of chronic anaemia by Hb, volume therapy, and hemodynamic is more imprecise compared with differential anaemia diagnostics not covered in the registry.

## Conclusions

The majority of severely injured patients are admitted without anaemia to the ER. Injury-associated moderate and severe anaemia is an independent predictor of mortality in severely injured patients.

## Abbreviations

AIS: Abbreviated Injury Scale
AUC: AUC—Akademie der Unfallchirurgie GmbH
ASA: American Society of Anesthesiologists Physical Status Score
ASS: Acetylsalicylic acid
CPR: Cardiopulmonary resuscitation
DGU: Deutsche Gesellschaft für Unfallchirurgie e.V
ER: Emergency room
GCS: Glasgow Coma Scale
Hb: Haemoglobin
ICU: Intensive care unit
ISS: Injury Severity Score
LOS: Length of hospital stay
MA: Moderate anaemia
NA: No or mild anaemia
NOAC: New oral anticoagulants
PCC: Prothrombin complex concentrate
PH: Pre-hospital
RBC: Red blood cell
RISC-2: Revised Injury Severity Classification II
SA: Severe anaemia
SIRS: Systemic inflammatory response syndrome
TR-DGU: TraumaRegister DGU^®^
